# Lotus Seedpod Extracts Reduced Lipid Accumulation and Lipotoxicity in Hepatocytes

**DOI:** 10.3390/nu11122895

**Published:** 2019-11-28

**Authors:** Yen-Tze Liu, Yen-Hsun Lai, Hui-Hsuan Lin, Jing-Hsien Chen

**Affiliations:** 1 Department of Family Medicine, Changhua Christian Hospital, No. 135 Nanhsiao Street, Changhua City 50006, Taiwan; ytliu411@gmail.com; 2 Department of Nutrition, Chung Shan Medical University, No. 110, Sec. 1, Jianguo N. Road, Taichung City 40201, Taiwan; ecfghswind@gmail.com; 3 Department of Medical Laboratory and Biotechnology, Chung Shan Medical University, No. 110, Sec. 1, Jianguo N. Road, Taichung City 40201, Taiwan; 4 Department of Clinical Laboratory, Chung Shan Medical University Hospital, No. 110, Sec. 1, Jianguo N. Road, Taichung City 40201, Taiwan

**Keywords:** non-alcoholic fatty liver disease, lipid accumulation, lipotoxicity, polyphenols, lotus seedpod, oleic acid, apoptosis

## Abstract

Non-alcoholic fatty liver disease (NAFLD) is closely associated with metabolic disorders, including hepatic lipid accumulation and lipotoxicity. Plant-derived polyphenols have attracted considerable attention in the prevention of NAFLD. Lotus seedpod, rich in polyphenols, is a traditional Chinese herbal medicine. Previous studies have showed that lotus seedpod possess radioprotective, antioxidant, anti-cancer, and anti-inflammatory activities. In this study, the in vitro hepatoprotective effect of lotus seedpod extract (LSE) and its main component epigallocatechin (EGC) was examined. Firstly, oleic acid (OA), an unsaturated fatty acid, was used to induce the phenotype of NAFLD in human hepatocytes, HepG2 cells. LSE dose-dependently improved the OA-induced viability loss of HepG2 cells. Non-cytotoxic concentrations of LSE or EGC abolished intracellular lipid accumulation and oxidative stress in the OA-treated cells. In addition, LSE and EGC showed a minor effect on autophagy, and potential in reducing OA-induced occurrence of apoptosis confirmed by morphological and biochemical features, including an increase in the formation of apoptotic bodies, the exposure of phosphatidylserine, and activation of caspases. Molecular data showed the anti-apoptotic effect of LSE might be mediated via downregulation of the mitochondrial pathway. Our data imply that EGC-enriched LSE potentially could be developed as an anti-NAFLD agent.

## 1. Introduction

Non-alcoholic fatty liver disease (NAFLD) is a clinical pathological illness that is distinguished with lipid accumulation without notable alcohol consumption, which is harmful for human health [1,2]. The pathogenesis of NAFLD is complex and multifactorial, mainly involving genetic, environmental, and metabolic factors [3]. NAFLD is promoted by obesity, dyslipidemia, impaired glucose tolerance, and is considered the hepatic metabolic dysfunctions induced by insulin resistance [4]. NAFLD is highly associated with chronic diseases, such as non-alcoholic steatohepatitis (NASH), hemoglobin A1c (HbA1c)-defined prediabetes, and type 2 diabetes (T2D) [4,5]. At present, the prevalence of NAFLD is about 20%–40% in the common population, which is a critical health menace for humans [6]. The pathogenesis of NAFLD remains perplexing, and the “two-hit” hypothesis has become a generally established view to guide researches [7]. The “first hit” is steatosis that mentions excessive triacylglycerol (TG) and free fatty acids (FFAs), which are generated by the disorder of hepatic lipid metabolism [8]. Mono-unsaturated fatty acids increase significantly in the FFA composition of the liver of patients with NAFLD, and oleic acid (OA) is the most important component [9]. The “second hit” promotes the forming of NAFLD followed with an inflammatory response, oxidative stress, and cellular apoptosis [8]. In a current study, it is considered the multiple-hit pathogenesis of NAFLD, meaning the relationship between NAFLD and metabolic syndrome, mainly insulin resistance and oxidative stress, is interaction [3]. Furthermore, oxidative stress is caused by reactive oxygen species (ROS) production, which has been known as a major factor related to NAFLD pathogenesis, and leads to cell membrane lipid peroxidation, mitochondrial dysfunction, and even apoptosis [2,10]. Autophagy-dependent degradation of organelles is a cellular pathway critical for keeping cell homeostasis, and organellar autophagy can be selective for the degradation of mitochondria (mitophagy) and lipid droplets (lipophagy) [11]. Lipophagy is presently regarded as an alternative pathway for lipid metabolism in hepatocytes, and the disability of lipophagy contributes to NAFLD development [12]. In addition, autophagy can lead to apoptosis by its association with the caspase-3 activation-dependent pathway [13]. Overall, the exact mechanism(s) by which autophagy and apoptosis influence NAFLD remain unknown.

Nelumbo nucifera Gaertn. (commonly known as lotus) is a perennial aquatic plant, grown and consumed in China, Japan, India, and some African countries. Nearly all parts of lotus, such as stems, leaves, flowers, and seeds, are eaten as foods, and also used for medicinal applications [14]. Further studies have shown that bioactive compounds, such as triterpenoid [15], alkaloid [16], polyphenolic acid [17], and flavonoids [18] can be extracted from different parts of this plant. Lotus seedpod is usually ignored and abandoned, apart from its occasional use as a traditional Chinese herbal medicine for removing bruises and hemostasis function [19]. It has been reported that lotus seedpod is rich in polyphenols and an important natural source of oligomers and polymers of catechin, which are also denominated procyanidins [19,20]. In recent years, procyanidins from lotus seedpod have been shown to possess antioxidant, anti-aging, anti-memory impairment, radioprotective, anti-glycative, and anti-inflammatory activities [20,21,22,23,24,25]. However, little information is available on the anti-lipid accumulation and anti-lipotoxic effect of lotus seedpod extract (LSE) in hepatocytes.

In this study, we investigated the hepatoprotective effect of LSE and its main components epigallocatechin (EGC) on human hepatocytes HepG2 induced by OA to mimic the influx of excess FFAs into hepatocytes, giving rise to hepatic steatosis [26]. HepG2 cells are a suitable in vitro model system for the study of polarized human hepatocytes. Due to their high degree of morphological and functional differentiation in vitro, HepG2 cells have been widely used as a model system for evaluating hepatic cells in lipid accumulation and lipotoxicity [27,28]. Our results indicated that LSE reduced intracellular lipid accumulation and lipotoxicity in OA-treated HepG2 cells by inhibiting apoptosis signals. 

## 2. Materials and Methods 

### 2.1. Preparation of LSE

Mature lotus seedpods of Nelumbo nucifera Gaertn. were obtained from Baihe District, Tainan City, Taiwan. The dried lotus seedpods (100 g) were macerated with hot water (95 °C, 4000 mL) for 2 h and the aqueous extract was evaporated under vacuum at −85 °C. The extracted solution was filtered, then lyophilized to obtain approximately 27 g of LSE and stored at −20 °C before use. The concentrations of total polyphenols and flavonoids contents of LSE were measured as described previously [25].

### 2.2. Cell Culture

HepG2 cells were purchased from the Bioresource Collection and Research Center (BCRC, Food Industry Research and Development Institute, Hsinchu, Taiwan, ROC). HepG2 cells were cultured in Minimal Essential Medium (MEM) with Earle’s balanced salt solution (EBSS). The cell culture medium was supplemented with 2 mM of L-glutamine, 1.5 g/L of sodium bicarbonate, 0.1 mM of non-essential amino acids (NEAA), 1.0 mM of sodium pyruvate, 100 U/mL of penicillin, 100 μg/mL of streptomycin, and 10% fetal bovine serum (FBS) at 37 °C in a humidified atmosphere of 5% CO_2_. Before treatment, cells were seeded at a density of 5 × 10^5^ onto 6-well plates for 24 h. To induce overloading of fatty acids, HepG2 cells at 70% confluence were exposed to OA (Sigma-Aldrich, St. Louis, MO, USA). OA/bovine serum albumin (BSA) complex was prepared as reported previously [6]. Stock solutions of 1 M of OA prepared in culture medium containing 10 μL/mL of BSA were conveniently diluted in the culture medium to obtain the desired final concentrations. The OA/BSA complex solution was sterile-filtered through a 0.22 μm pore membrane filter and stored at −20 °C.

### 2.3. Trypan Blue Dye Exclusion Assay

The trypan blue dye exclusion assay was used to evaluate the effect of the test drugs on cell viability, as described previously [25], and to determine the non-cytotoxic concentrations. Cells were seeded at a density of 10^5^ cells/mL and treated with OA (0.6 mM) in the presence or absence of LSE or EGC at various concentrations for 24 h or 48 h. Afterward, the cells were stained with trypan blue, and live cells were enumerated to determine % cell growth. 

### 2.4. Oil Red O Staining 

HepG2 cells (10^5^ cells/mL) were seeded in a 6-well culture plate and cultured with OA (0.6 mM), with or without LSE (2.5, 5, and 10 μg/mL) and EGC (4 μM) for 48 h. After the medium was removed, cells were washed twice with phosphate buffered saline (PBS), and then fixed with 4% paraformaldehyde in PBS (pH 7.3) for 30 min. The fixed cells were washed with distilled water and then stained with oil red O (Sigma-Aldrich, St. Louis, MO, USA) for 15 min. The stained cells were observed under a microscope, and their pictures were taken at 200×. Furthermore, it can be used for quantifying the extent of the cell through extracting the dye with isopropanol and the absorbance spectrophotometrically at 490 nm (OD 490 nm). All results were presented as the mean ± SD of three independent experiments. Values were expressed as a percentage change compared with the control group, which was set as 100%.

### 2.5. Nile Red Stain

HepG2 cells (1 × 10^5^ cells/mL) were seeded in a 6-well culture plate and cultured with OA (0.6 mM), with or without LSE (2.5, 5, and 10 μg/mL) and EGC (4 μM) for 48 h. After the medium was removed, cells were washed twice with PBS and then fixed with 4% paraformaldehyde in PBS (pH 7.3) for 30 min. The fixed cells were washed with distilled water and then stained with Nile red (1 μg/mL) (Sigma-Aldrich, St. Louis, MO, USA) for 5 min. The fluorescence intensity of each sample was measured immediately by Muse™ Cell Analyzer (Millipore, Hayward, CA, USA) at an excitation wavelength of 488 nm and an emission wavelength of 550 nm.

### 2.6. Reactive Oxygen Species (ROS) Assay

Intracellular production of ROS, namely hydrogen peroxide, was measured using dichlorofluorescin diacetate (DCFH-DA) (Enzo Life Sciences, Inc., Farmingdale, NY, USA). Cells (at 10^5^ cells/well) from each experimental condition were stained with DCFH-DA (2 µM) at 37 °C for 30 min. ROS production of cells was evaluated by flow cytometry (Becton Dickinson, Warwick, RI, USA). Values were expressed relative to the fluorescence signal of the control.

### 2.7. Acridine Orange (AO) Staining

The volume of the cellular acidic compartment, as a marker of autophagy, was examined by staining with lysosomotropic agent AO (Sigma-Aldrich, St. Louis, MO, USA). AO was seen to move freely across a biological membrane, and accumulate in the acidic compartment where it was observed as fluorescence bright red. After treatments, the cells were stained with AO (1 µg/mL) for 15 min at room temperature in the dark. Acidic vesicular organelles were obtained with a fluorescent microscope and photographed. The extent of acidic vesicular organelles formation was further quantified by a FACScan with the CELLQuest program (BD Biosciences, San Jose, CA, USA). AO uptake was analyzed by flow cytometry, with the control group designated as 100%.

### 2.8. 4,6-Diamidino-2-Phenylindole (DAPI) Staining

The characteristic of apoptotic cell morphology was assayed by fluorescence microscopy of DAPI-stained cells. After treatments, the monolayer of cells was washed with PBS and fixed in 4% paraformaldehyde for 30 min at room temperature. The fixed cells were permeabilized with 0.2% Triton X-100 in PBS three times and then incubated with 1 µg/mL of DAPI solution for 30 min. After washing with PBS three times, the nuclei (intensely stained, fragmented nuclei, and condensed chromatin) of apoptotic cells were observed under 400× magnification using a fluorescent microscope with a 340/380 nm excitation filter. The percentage of apoptosis was calculated as the proportion of apoptotic cells relative to total cells counted. At least three separate experiments were conducted, and at least 300 cells were counted for each experiment.

### 2.9. Annexin V-Fluorescein Isothiocyanate (FITC) and 7-Amino-Actinomycin (7-AAD) Double Staining

Annexin V-FITC detects the translocation of phosphatidylinositol from the inner to the outer cell membrane during early apoptosis, and 7-AAD can enter the cell in late apoptosis or necrosis [29]. After treatments, the cells were washed twice with cold PBS and resuspended in 1× binding buffer (BD Bioscience, Franklin Lakes, NJ, USA). Then, 100 μL of solution was transferred to 5 mL culture tubes. These cells were stained with 5 μL annexin V-FITC and 10 μL 7-AAD (BD Bioscience, San Jose, CA, USA), gently vortexed, and incubated at ambient temperature for 15 min in the dark. Following this, 400 μL 1× binding buffer was added to each tube and analyzed within an hour using a flow cytometry method and data analyzed by Muse ExpressPro software (Millipore, Hayward, CA, USA). For each measurement, at least 20,000 cells were counted.

### 2.10. Mitochondrial Membrane Depolarization Assay

The alteration of mitochondrial membrane potential (ΔΨm) in HepG2 cells were analyzed through a JC-1 staining assay kit according to the manufacturer’s instructions (Beyotime, Hangzhou, China). Briefly, after incubating with OA (0.6 mM), with or without LSE (2.5, 5, and 10 μg/mL) and EGC (4 μM) for 48 h, the cells were then rinsed with PBS and stained with JC-1 (20 μg/mL) at 37 °C in the dark for 30 min. After rinsing with a staining buffer twice, the samples were detected by flow cytometry.

### 2.11. Western Blot Analysis

Western blotting was performed as previously described [29,30]. In short, the cell lysates were denatured in a sample buffer containing sodium dodecyl sulfate (SDS), and equal amounts of total protein were separated on 8%–15% SDS-poly-acrylamide gels and transferred to nitrocellulose membranes. After blocking with 5% nonfat dry milk, the membranes were incubated overnight at 4 °C with the primary antibodies as indicated. The following antibodies were used: against active-caspase-3 (sc-271028), active-caspase-8 (sc-5263), active-caspase-9 (sc-133109), cleaved cleaved-poly adenosine diphosphoribose polymerase 1 (PARP-1) (sc-56196), cytochrome c (sc-13156), cytochrome c oxidase IV (COX IV) (sc-376731), Bcl-2 (sc-492), and Bax (sc-526) were purchased from Santa Cruz Biotechnology Inc. (Santa Cruz, CA, USA). Anti-â-actin was purchased from Sigma Chemical Co. (St. Louis, MO, USA), and these antibodies against LC3, Atg5/12 conjugate, and Beclin-1 were purchased from Novus Biological Inc (Littleton, CO, USA). The following day, the membranes were incubated with the appropriate horseradish peroxidase-conjugated secondary antibodies, and detection was performed using enhanced chemiluminescence (ECL) reagent (Amersham Life Science).

### 2.12. Statistical Analysis

Three or more separate experiments were performed. Data were reported as mean ± standard deviation (SD) of three independent experiments. Student’s *t*-test was used for the analysis between two groups with only one factor involved. For the experiments of dose response of LSE, one-way analysis of variance (ANOVA) with post-hoc Dunnett’s test was used to calculate the *p*-value for each dose treatment compared to the control (OA alone, without LSE). Regression was used to test the *p*-value of the dependency of a parameter to dosage. Significant differences were established at *p* < 0.05.

## 3. Results

### 3.1. LSE Attenuated the Cytotoxic Effect of OA in HepG2 cell

**Figure 1:**
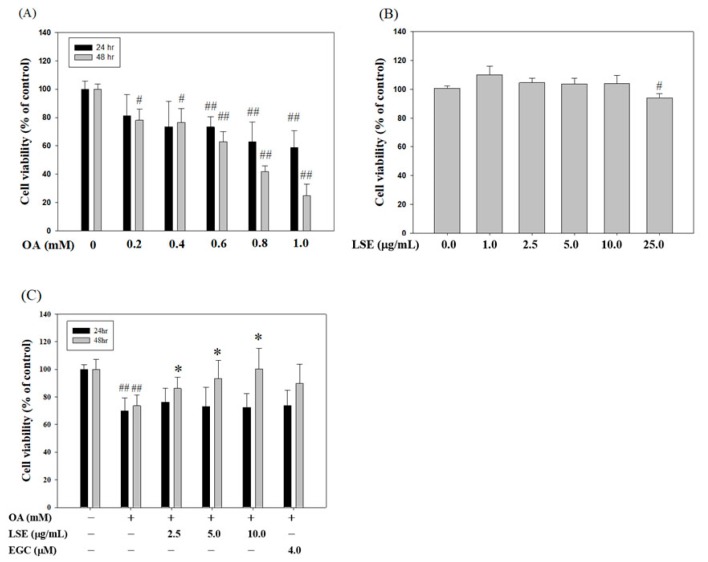
Effects of oleic acid (OA) or lotus seedpod extract (LSE) alone and in combination on HepG2 cell viability. (**A**) HepG2 cells were treated with various concentrations (0–1.0 mM) of OA for 24 h or 48 h. (**B**) HepG2 cells were treated with various concentrations (0–25 μg/mL) of LSE for 48 h. (**C**) HepG2 cells were treated with or without OA (0.6 mM) in the presence or absence of LSE (2.5, 5, and 10 μg/mL) or epigallocatechin (EGC) (4 μM) for 24 h or 48 h. The cell viability was assayed by trypan blue dye exclusion assay. The quantitative data were presented as mean ± SD of three independent experiments. ^#^ *p* < 0.05, ^##^ *p* < 0.01 compared with control via Student’s *t*-test. * *p* < 0.05 compared with the OA group via one-way ANOVA with post-hoc Dunnett’s test. +: added. -: non-added.

HepG2 cell survival was tested following incubation with a range of doses of OA (from 0.2 to 1.0 mM) for 24 h and 48 h, and it was found that OA at higher concentrations (lower than 0.6 mM) dose- and time-dependently decreased cell viability (Figure 1A). After a 48 h incubation period, the concentration of OA on the inhibition of 50% of HepG2 cells viability (IC_50_) was about 0.8 mM, whereas the dose of 0.6 mM of OA reduced nearly 30% of cell viability (Figure 1A). In addition, to demonstrate that LSE is an inhibitor of OA-induced cytotoxicity and lipid deposition, we excluded the effect of LSE alone on HepG2 cell growth by trypan blue dye exclusion assay showing that the cell viability was not significantly altered by the treatment of LSE at doses of <25 μg/mL (Figure 1B). As shown in Figure 1C, the decreases were increased in the cells incubated with combinations of OA and increasing concentrations of LSE at 2.5, 5, and 10 μg/mL or EGC at 4 μM (the concentration of EGC in LSE at 10 μg/mL was approximately 1.26 μg/mL, which was equivalent to about 4 μM [25]) for 48 h, when compared to the OA alone group. It is worth noting the combination of OA and LSE indicated significant antagonistic efficacy, especially in the dose of 10 μg/mL of LSE, which almost completely blocked the OA-inhibited cell growth. The doses of the combination were selected for all further studies.

### 3.2. Effects of LSE on the OA-Induced Intracellular Lipid Accumulation

**Figure 2:**
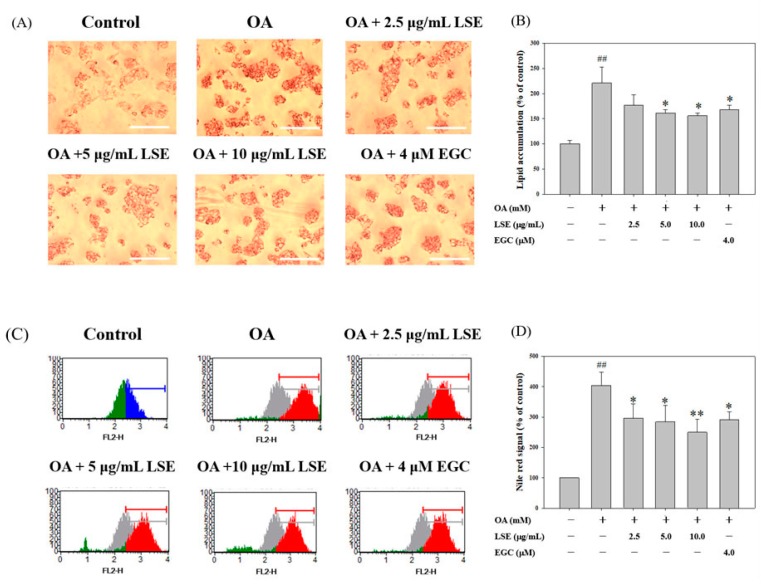
Effects of LSE or EGC on the OA-induced intracellular lipid accumulation. HepG2 cells were treated with 0.6 mM of OA in the presence or absence of LSE (2.5, 5, and 10 μg/mL) or EGC (4 μM) for 48 h. (**A**) After incubation, the cells were stained with oil red O and then observed under a microscope (100× magnification; scale bar, 50 μm). The red droplets accumulated in the cells were indicated as the stained lipid. (**B**) Quantitative analysis of intracellular lipid accumulation was measured at 490 nm after extraction of the oil red O stain. (**C**) Quantification of intracellular fat content with flow cytometry, as assessed by Nile red staining. (**D**) The quantitative data are presented as mean ± SD of two independent experiments. ^##^ *p* < 0.01 compared with control via Student’s *t*-test. * *p* < 0.05, ** *p* < 0.01 compared with the OA group via one-way ANOVA with post-hoc Dunnett’s test.

OA is a monounsaturated fatty acid in which inadequate metabolism induces an adverse cellular response termed lipotoxicity [9,31]. Lipotoxicity is a metabolic syndrome that is caused by the accumulation of lipids in the liver and leads to cellular dysfunction and death [32]. As shown in Figure 2, the lipid contents of HepG2 cells were examined by oil red O (Figure 2A,B) and Nile red staining (Figure 2C,D), respectively. When the cells were treated by OA at 0.6 mM, cellular steatosis was successfully induced with a statistical difference in the absorbance compared with the control group (Figure 2B). The data of Figure 2B also showed that treatments of LSE dose-dependently inhibited intracellular lipid accumulation. These results were further confirmed by Nile red staining. OA treatment alone caused a significant increase in lipid accumulation (Figure 2C). As shown in Figure 2D, the OA-induced increases in levels of lipid accumulation were reduced by 35.5%, 39.1%, and 50.7% in 2.5, 5, and 10 μg/mL of LSE, respectively, as compared to OA treatment. It is noteworthy that the inhibitory effect of EGC at 4 μM on lipid accumulation was similar to LSE at 5–10 μg/mL (Figure 2).

### 3.3. Effects of LSE on OA-Induced an Increase in ROS Content in HepG2 cells

**Figure 3:**
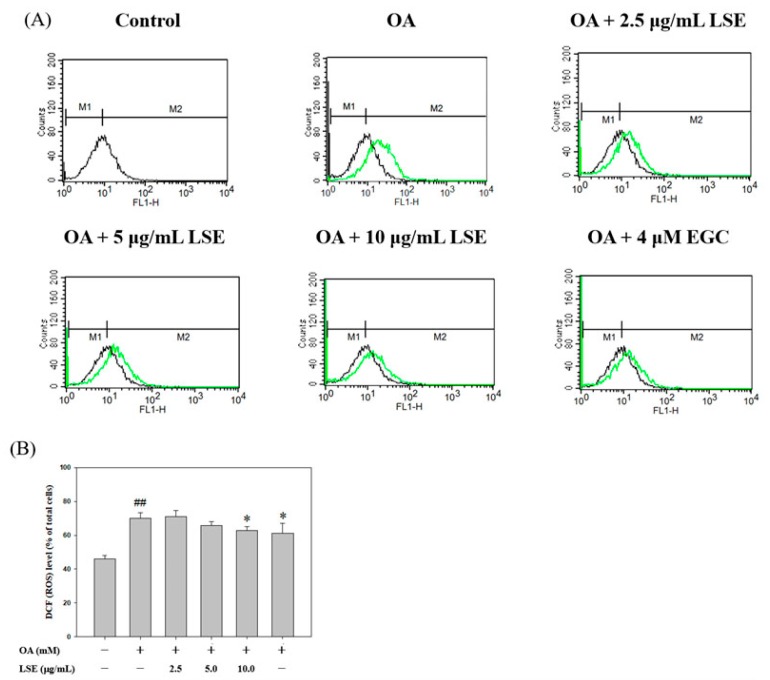
Effects of LSE or EGC on OA induced an increase in reactive oxygen species (ROS) content in HepG2 cells. HepG2 cells were treated with 0.6 mM of OA in the presence or absence of the indicated concentrations of LSE (2.5, 5, and 10 μg/mL) or EGC (4 μM) for 48 h. (**A**) The ROS content was assayed by dichlorofluorescin diacetate (DCFH-DA) staining with flow cytometry. M1: DCF-negative cells. M2: DCF-positive cells. (**B**) ROS levels were presented as a percentage of DCF-positive cells divided by the total number of cells. The quantitative data were presented as mean ± SD of three independent experiments. ^##^ *p* < 0.01 compared with control via Student’s *t*-test. * *p* < 0.05 compared with the OA group via one-way ANOVA with post-hoc Dunnett’s test.

ROS, as a potent oxidant in cells, has been reported previously to play an important role in the development of NAFLD via the promotion of neutral lipid accumulation [2]. To investigate the free radical scavenging effect of LSE resulting from cellular steatosis, the ROS generation (DCF fluorescence) following the LSE treatments in the OA-stimulated cells was examined (Figure 3A). The results showed that OA significantly increased the fluorescence of intracellular ROS production, but LSE (10 μg/mL) or EGC (4 μM) protected against such ROS generation (Figure 3B).

### 3.4. Effects of LSE on OA-Induced HepG2 Cell Autophagy

**Figure 4:**
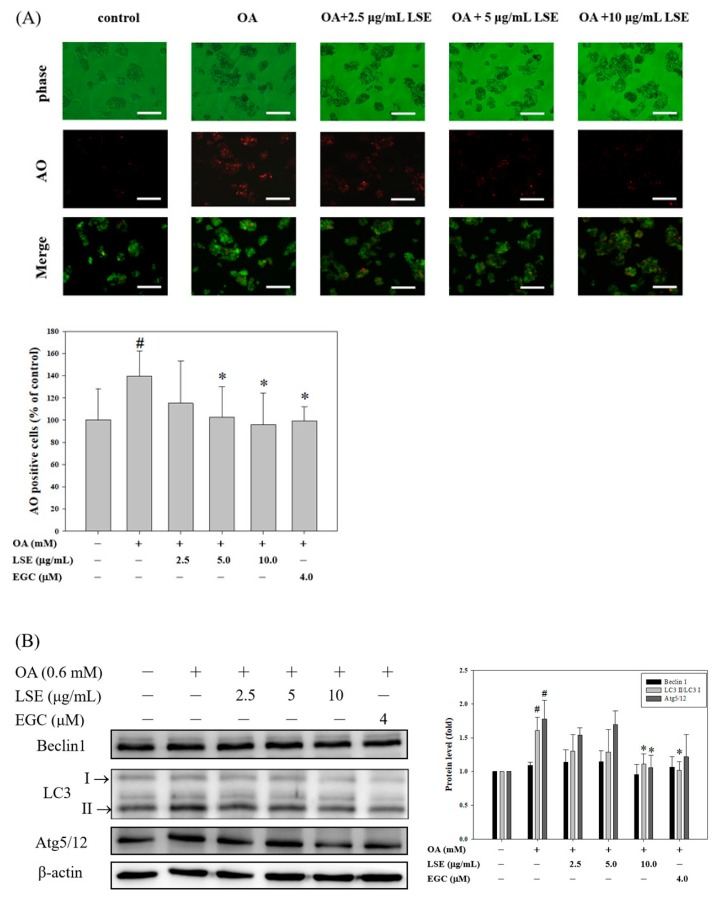
Effects of LSE or EGC on OA-induced HepG2 cell autophagy. HepG2 cells were treated with 0.6 mM of OA in the presence or absence of LSE (2.5, 5, and 10 μg/mL) or EGC (4 μM) for 48 h. (**A**) Autophagic cells were assayed by AO staining. Panels show (from up to down) phase-contrast microscopy (upper), AO staining (middle), and merge image (lower). Autophagic values were calculated as the AO-positive cells in each random field (>100 cells) and represented as mean ± SD (*n* = 3) of three independent experiments. Images were taken at 100× magnification; scale bar, 30 μm. (**B**) The protein levels of Beclin 1, LC3-II/LC3-I, and Atg5/12 were analyzed by Western blotting. β-actin served as an internal control. The quantitative data were presented as mean ± SD of three independent experiments. ^#^ *p* < 0.05 compared with control via Student’s *t*-test. * *p* < 0.05 compared with the OA group via one-way ANOVA with post-hoc Dunnett’s test.

Autophagy is an evolutionarily conserved intracellular degradation function that is important for liver cell homeostasis [33]; however, the mechanism remains incompletely understood. The molecular events activating the autophagic mechanism after OA with or without LSE or EGC treatments were further studied. First, by the AO staining method, control cells displayed green fluorescence in cytoplasm and nucleolus, but cells exposed to OA at 0.6 mM for 48 h showed increases in red fluorescent dots presented in cytoplasm, indicating the formation of acidic autophagolysosomal vacuoles (Figure 4A). As compared with the OA-treated group, the addition of LSE or EGC to the OA-treated cells slightly reduced autophagy, which could be an antagonist in the formation of autophagic vacuoles (Figure 4A). To confirm the autophagy-inhibitory role of LSE or EGC on OA-treated cells, the protein expressions of autophagy-related proteins, Beclin1, LC3, and Atg5/12, were determined by Western blotting. LC3 processing, namely the increased ratio of LC3-II/LC3-I, was induced in the cells treated with 0.6 mM of OA for 48 h, indicating that OA induced autophagy in HepG2 cells (lane 2, Figure 4B). It was also found that the cellular LC3-II/LC3-I ratio and Atg5/12 level, but not Beclin-1, were slightly and significantly decreased after being treated with a higher dose of LSE (10 μg/mL) or EGC. These results indicated that LSE can slightly inhibit the OA-induced HepG2 cell autophagy.

### 3.5. Effects of LSE on OA-Induced HepG2 Cell Apoptosis

**Figure 5:**
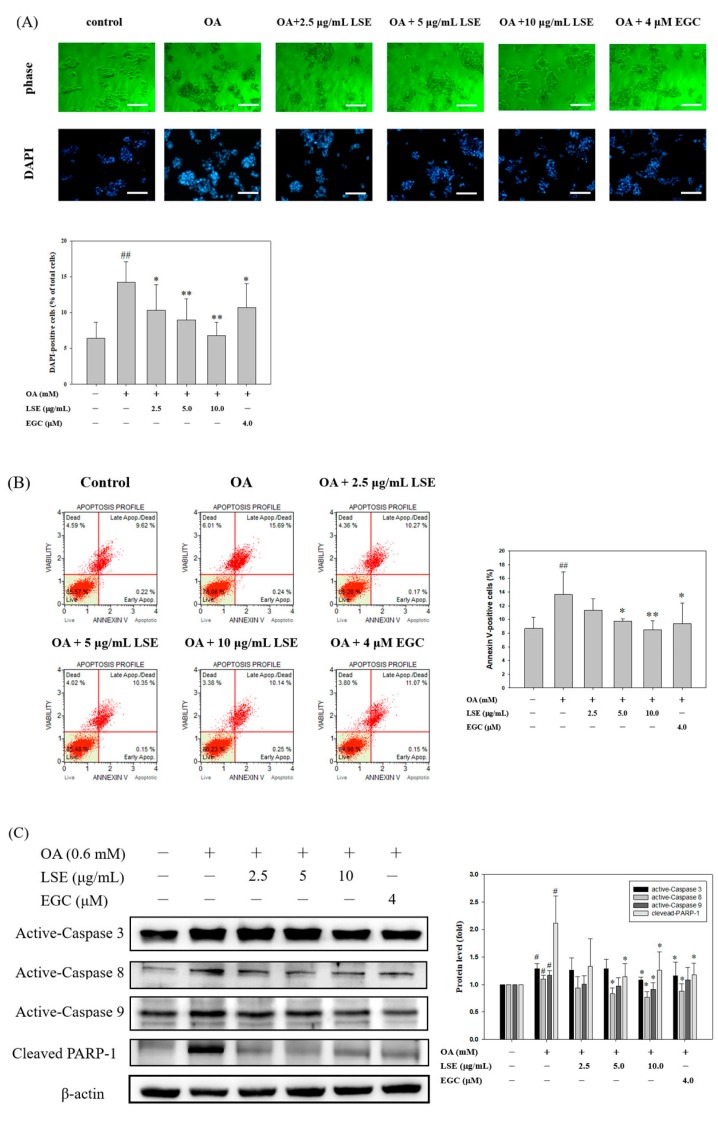

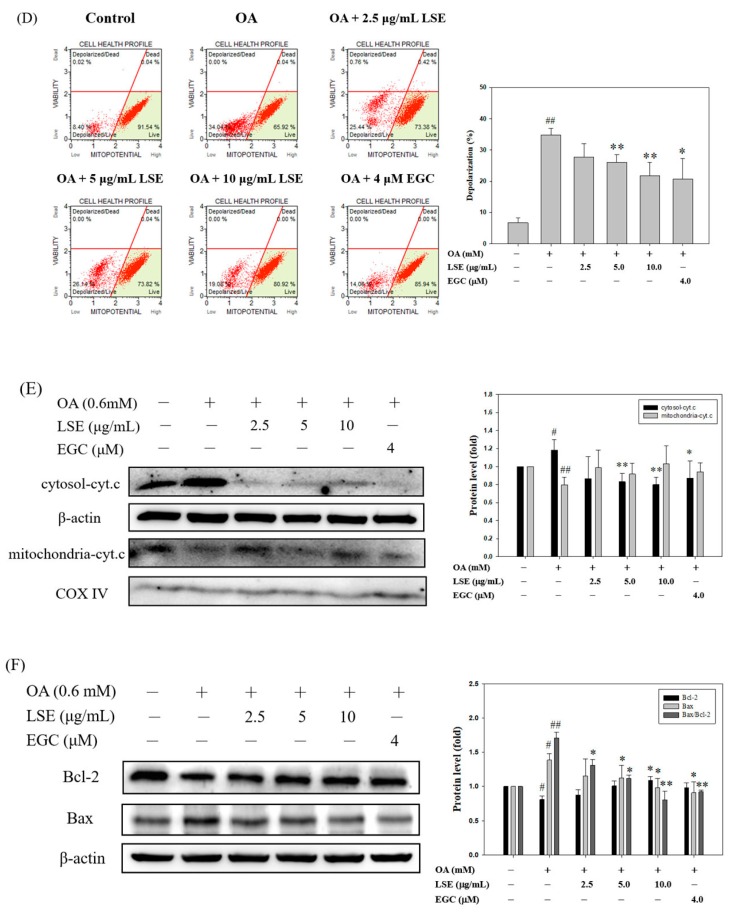
Effects of LSE or EGC on OA-induced HepG2 cell apoptosis. HepG2 cells were treated with or without OA (0.6 mM) in the presence or absence of LSE (2.5, 5, and 10 μg/mL) or EGC (4 μM) for 48 h. (**A**) The apoptotic cells were assayed by the 4,6-diamidino-2-phenylindole (DAPI) assay. Apoptotic values were presented as the percentage of apoptotic cells divided by the total number of cells. Images were taken at 100× magnification; scale bar, 30 μm. (**B**) Quantification of early and late apoptosis cells was analyzed by flow cytometry using annexin V-fluorescein isothiocyanate (FITC) and 7-amino-actinomycin (AAD) double staining. The proportion of annexin V-positive cells is represented as mean ± SD (*n* = 3) of three independent experiments. (**C**) The protein levels of active-caspase-3, active-caspase-8, active-caspase-9, and cleaved PARP-1 were analyzed by Western blotting. β-actin served as an internal control. (**D**) The mitochondrial membrane depolarization was assayed by JC-1 staining with flow cytometry. Depolarization levels were presented as percentage of depolarization cells divided by the total number of cells. (**E**) The protein levels of cytosol and mitochondria cytochrome c (cyt. c) were analyzed by Western blotting. β-actin and COX IV (cytochrome c oxidase IV) respectively served as an internal control of cell cytosol and mitochondria. (**F**) The protein levels of Bcl-2 and Bax were analyzed by Western blotting. β-actin served as an internal control. The quantitative data were presented as mean ± SD of three independent experiments. ^#^ *p* < 0.05, ^##^ *p* < 0.01 compared with control via Student’s *t*-test. * *p* < 0.05, ** *p* < 0.01 compared with the OA group via one-way ANOVA with post-hoc Dunnett’s test.

To hypothesize that LSE may be involved in OA-induced HepG2 cell apoptosis, further experiments were conducted. The apoptosis induction in the exposure of HepG2 cells to OA was detected by DAPI staining and annexin V-FITC analysis. Exposure of HepG2 cells to OA resulted in morphologic alterations characteristic of apoptosis, including cell shrinkage, nuclear condensation, and fragmentation, but LSE protected against such injuries in a dose-dependent manner (Figure 5A). The proportion of apoptotic cells was quantified by DAPI staining. After exposure to OA for 48 h, the percentage of DAPI-positive cells, representing DNA fragmentation, increased by about 15%. In LSE or EGC-cotreated cells, the proportion of DAPI-positive cells decreased (Figure 5B). Flow cytometry was used to examine annexin V-FITC and 7-AAD double staining, and it revealed a significant shift in annexin V-positive cells in the OA group, as shown in Figure 5B. The OA-induced proportion of annexin V-positive cells was also decreased by LSE or EGC, which appeared in similar variation tendency to DAPI staining. 

To investigate whether the LSE protection against OA occurred because it inhibited apoptotic pathways, we further studied the changes in expressions of active-caspase-3 (p17), active-caspase-8 (p18), active-caspase-9 (p35), and cleaved-poly (ADP-ribose) polymerase 1 (c-PARP-1), markers of apoptosis, in the HepG2 cells (Figure 5C). Stimulation with OA significantly induced the activation of these caspases and cleavage of PARP-1, compared to that of the control group. After exposure to OA for 48 h, LSE or EGC treatments inhibited these protein expressions, with higher concentrations being more effective (Figure 5C). One of the most significant events in apoptosis is mitochondrial dysfunction. Loss of mitochondrial transmembrane potential (MTP) elicits the mitochondrial release of cytochrome c (cyt. c) to cytosol [34]. In Figure 5D,E, OA-induced mitochondrial membrane depolarization of HepG2 cells and release of cyt. c from mitochondria to cytosol were decreased by LSE or EGC. Furthermore, it is evident that the pro-apoptotic protein Bax plays an essential role in the onset of MTP changes and induces cyt. c release, which is inhibited by the anti-apoptotic protein Bcl-2 [34]. Previous studies have also showed that the Bax/Bcl2 ratio is increased during apoptosis [34,35]. As shown in Figure 5F, OA-induced increase in the Bax/Bcl2 ratio was also revered by LSE or EGC. Our results implied that LSE and EGC can decrease OA-induced HepG2 cell apoptosis involving the mitochondrial pathway.

## 4. Discussion

**Figure 6:**
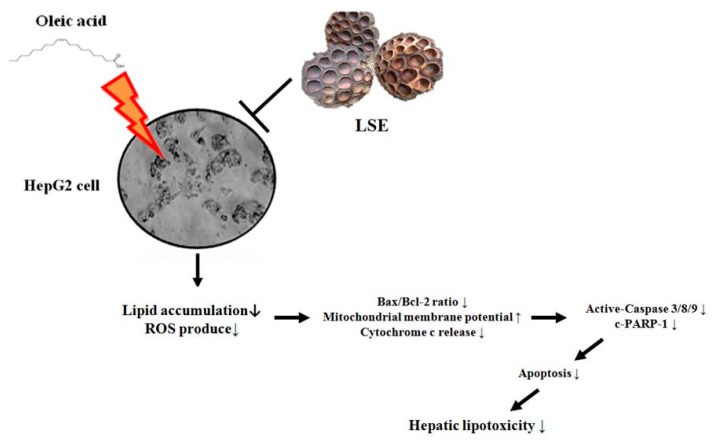
Overview of pathways for LSE inhibited the OA-induced hepatic lipotoxicity.

NAFLD is a chronic liver disease described by hepatic steatosis, and oxidative stress is considered as an important factor in NAFLD pathogenesis. To date, inhibition of hepatic lipid accumulation and oxidative stress is an attractive strategy for clinical therapy of NAFLD. Previous studies have addressed that palmitic acid (PA), a saturated non-esterified fatty acid, is a considerable cytotoxic agent on hepatoma cell lines and human hepatocyte primary cultures [36]. Studies have also shown OA to be less toxic than PA and to prevent/attenuate PA hepatocytes’ toxicity in steatosis models in vitro [37]. Therefore, to avoid that differential or opposite effect of OA and PA on lipid accumulation and apoptosis, a 48-h treatment of OA at a lower dose of ≤0.6 mM, which has shown less and significant cytotoxicity to HepG2 cells, was then applied in all subsequent experiments. As expected, our data showed that the number of lipid droplets and the content of the oil red O lipid were increased significantly in the OA-induced HepG2 cells, indicating the hepatic steatosis model in vitro was successfully established. By spectroscopic and biochemical methods, we further concluded that LSE reduced OA-induced hepatic lipotoxicity through inhibition of lipid accumulation and ROS production. The possible inhibitory pathways of LSE on OA-mediated lipotoxicity were primarily interfering apoptotic pathways, including decreases in (i) Bax/Bcl2 ratio; (ii) MTP elicits the mitochondria release of cyt. c to cytosol; (iii) protein levels of active-caspase-3/8/9 and c-PARP-1. To summarize, we proposed a schematic presentation of possible mechanisms for the inhibitory effect of LSE on the OA-induced hepatic lipotoxicity of HepG2 cells (Figure 6).

Many studies indicated that ROS plays an important role in oxidative stress [38,39]. Increased ROS production induces mitochondrial dysfunction, which increases steatosis, apoptosis, and inflammation in the liver [39]. This course can have a lethal aftermath and lead to liver cirrhosis and carcinoma [40]. It has been informed that EGC enriched LSE as an anti-inflammatory agent in Lipopolysaccharides-stimulated HepG2 cells and mice [25]. Recent evidences have focused on the control for NAFLD pathogenesis using micronutrient antioxidants and/or dietary components, such as vitamins and polyphenols [41,42,43]. In the literature, polyphenols are a heterogeneous class of plant-derived compounds, with some confirmed hepatoprotective effects [44]. Vitamins are known to interact with ROS to impede the extension of radical reactions in oxidative stress-related circumstances [45]. Our results showed that polyphenols-enriched LSE reduced the ROS production in OA-induced lipid accumulation and oxidative stress, which is consistent with previous studies.

Past studies have shown that autophagy is harmed in patients with NAFLD [46,47]. Many factors involved in endoplasmic reticulum stress, insulin resistance, and mitochondrial dysfunction have been demonstrated to conduce to the exacerbation of NAFLD and are related to the disability of autophagy in the context of NAFLD [47]. Thus, the disability of autophagy is related to lipotoxicity and the exacerbation of NAFLD. In this study, the OA-induced autophagy-related protein (LC3-II/LC3-I ratio and Atg5/12 conjugation) expressions were slightly reduced by LSE or EGC treatments. These results indicate the autophagy pathway is not majorly involved in LSE-mediated inhibition of hepatic lipotoxicity in HepG2 cells treated with OA.

Oxidative stress and mitochondrial dysfunction have been known to be concerned with the development of various metabolic diseases. Excessive ROS production and mitochondrial dysfunction are usually found in hepatocytes overloaded with FFAs [48,49]. Hence, agents with the capability to mitigate oxidative stress and mitochondrial dysfunction also have great potential in precaution of hepatic damage or other metabolism disorders. In this study, we found that LSE decreased OA-induced ROS generation, and improved mitochondrial function by decreasing the loss of MTP. Mitochondrial dysfunction results in the exit of pro-apoptotic mediators, such as cyto. c and second mitochondrial activator of apoptosis, into the cytosol where they accelerate the activation of effector caspases culminating in cellular destruction [50]. Mitochondrial dysfunction is regulated by proteins of the Bcl-2 family [35,50]. As data indicates that the OA-induced mitochondrial cyt. c releasing to cytosol and Bax/Bcl2 ratio was also decreased by LSE. However, the mechanism of LSE-regulated mitochondrial pathways of apoptosis is in need of further study. Understanding the signaling pathway involved in the process will hopefully shed light on strategies to improve therapeutic approaches to NAFLD chemical therapy. LSE represents an accessible possible source of polyphenols useful for preparation of food supplement. Our findings indicate that LSE could be developed as potent anti-NAFLD agents and natural healthy foods for the management of hepatic lipid accumulation and lipotoxicity.

## 5. Conclusions

In summary, our study offers a latent molecular mechanism considering the hepatoprotective effects of lotus seedpod, showing that LSE supplement may serve as a different treatment for hepatic lipid accumulation and lipotoxicity, and may be beneficial in the prevention and/or treatment of human NAFLD.
